# Microbial metal resistance and metabolism across dynamic landscapes: high-throughput environmental microbiology

**DOI:** 10.12688/f1000research.10986.1

**Published:** 2017-06-29

**Authors:** Hans Carlson, Adam Deutschbauer, John Coates

**Affiliations:** 1 Environmental Genomics and Systems Biology, Lawrence Berkeley National Lab, Berkeley, CA, USA; 2 Department of Plant and Microbial Biology, University of California, Berkeley, CA, USA

**Keywords:** microbial activity, inorganic compounds, metal-metabolism interactions

## Abstract

Multidimensional gradients of inorganic compounds influence microbial activity in diverse pristine and anthropogenically perturbed environments. Here, we suggest that high-throughput cultivation and genetics can be systematically applied to generate quantitative models linking gene function, microbial community activity, and geochemical parameters. Metal resistance determinants represent a uniquely universal set of parameters around which to study and evaluate microbial fitness because they represent a record of the environment in which all microbial life evolved. By cultivating microbial isolates and enrichments in laboratory gradients of inorganic ions, we can generate quantitative predictions of limits on microbial range in the environment, obtain more accurate gene annotations, and identify useful strategies for predicting and engineering the trajectory of natural ecosystems.

## Life, the universe and everything

**Figure 1.**
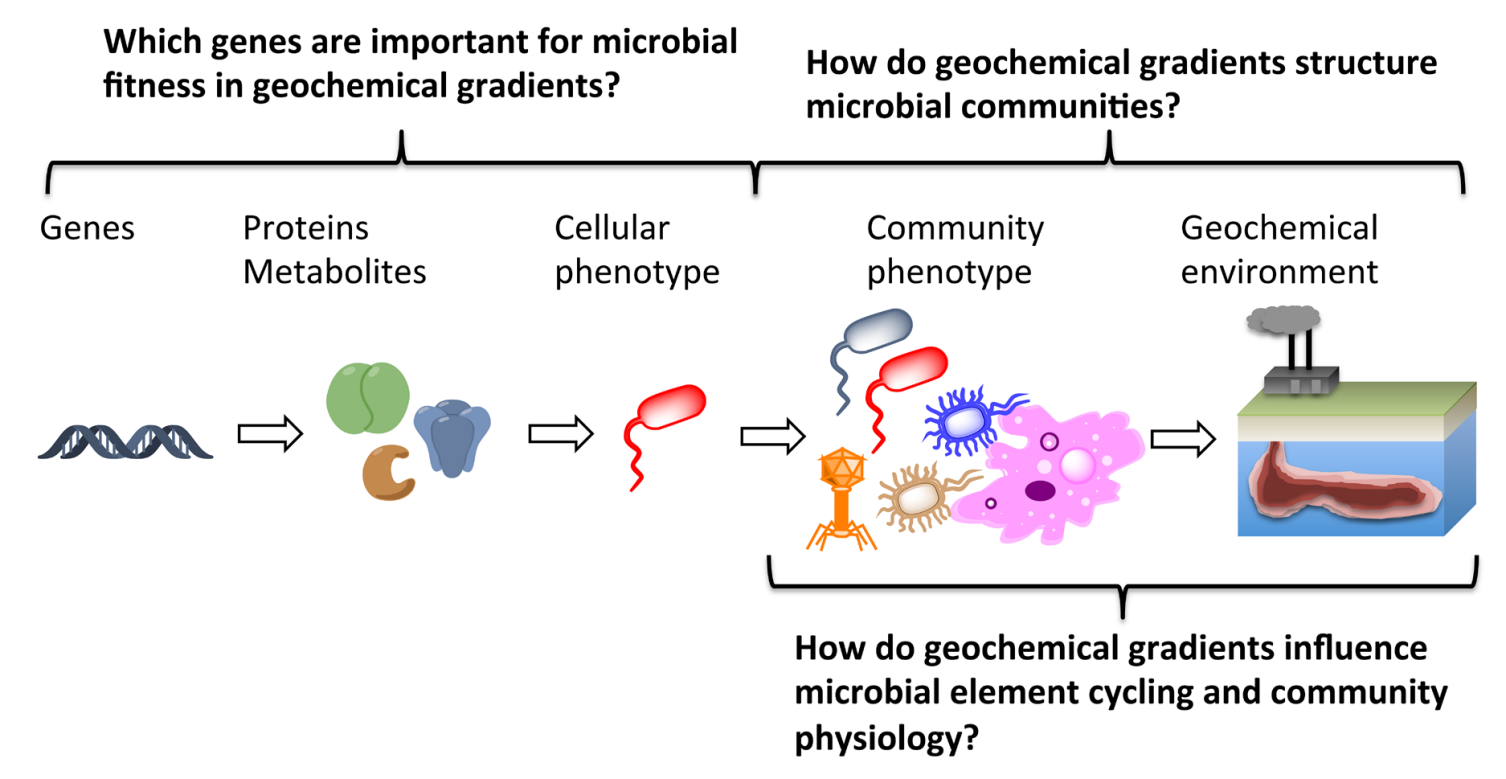
Understanding mechanisms whereby microorganisms survive in geochemical gradients is a central goal of environmental microbiology. Understanding mechanisms whereby microorganisms survive in geochemical gradients is a central goal of environmental microbiology.

In the book, *The Hitchhiker’s Guide to the Galaxy*, the Earth is described as “a computer of such infinite and subtle complexity that organic life itself shall form part of its operational matrix” ^1^. Understanding the workings of the Earth as a deterministic computational entity remains a tantalizing object, and characterizing the relationship between organic life and the rest of the “operational matrix” (that is, inorganic geochemistry) is a central theme in the environmental sciences. Although careful studies have yielded insights into how physical and chemical laws influence microbial fitness and function in response to some environmental parameters, a major challenge lies in scaling laboratory experiments to landscape-wide predictions of gene and microbial fitness ( Figure 1).

## Recontextualizing environmental isolates through high-throughput microbial physiology and genetics

**Figure 2.**
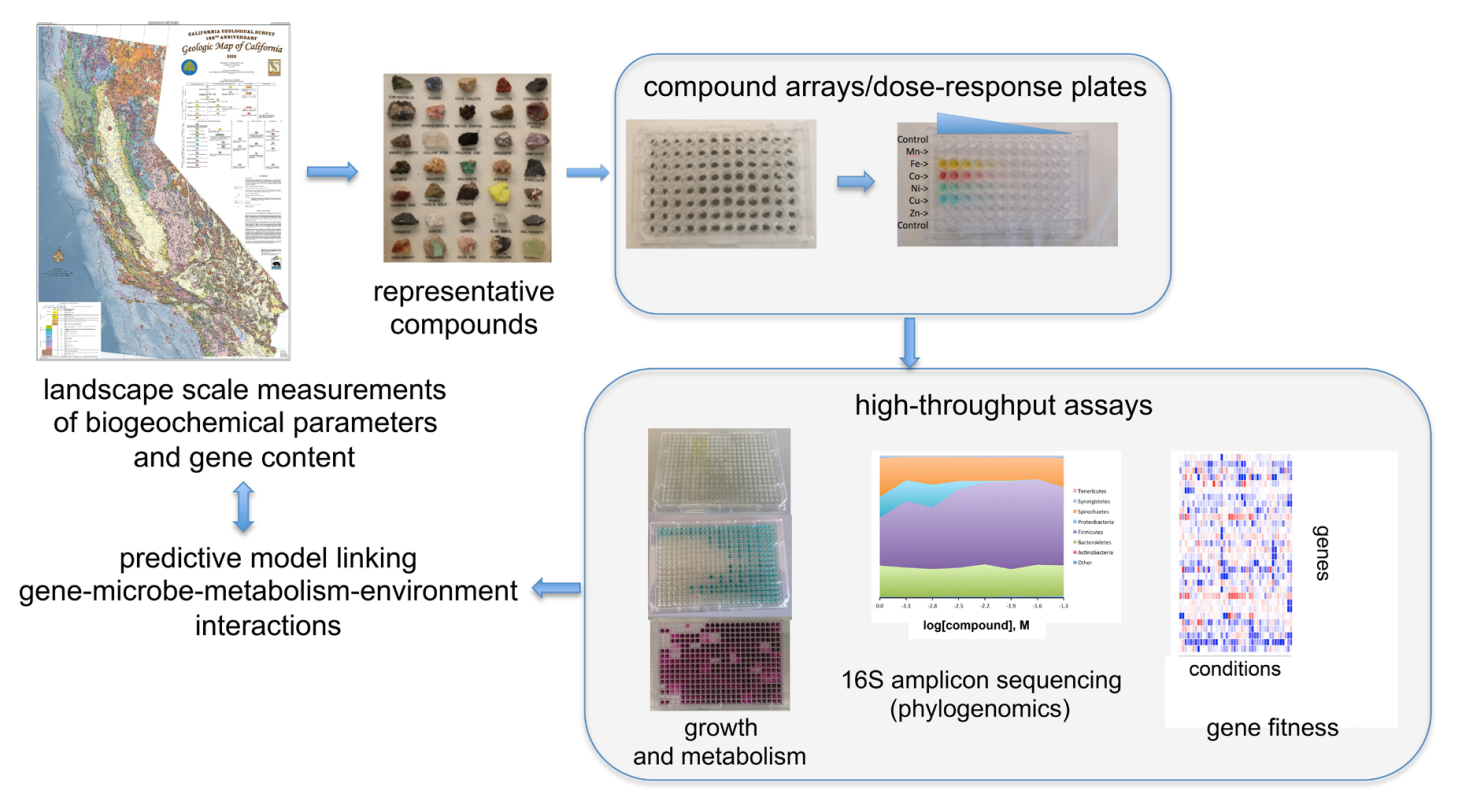
High-throughput cultivation pipelines can be used to evaluate gene-microbe-metabolism fitness in response to gradients of naturally occurring inorganic compounds. Measurements of metal content across rock, soil, and water samples can be obtained, and landscape-scale elemental maps can be constructed. Mineral samples and metal ions can be arrayed in microplates, and tagged-transposon pool assays, 16S amplicon sequencing, and metabolism-specific colorimetric assays can be employed to quantify the influence of concentrations of various metals on gene-microbe-metabolism fitness. Linking landscape-scale measurements of geochemistry to high-throughput laboratory measurements of microbial activity in response to geochemistry will enable higher-resolution biogeochemical models.

Great strides have been made in our ability to characterize the molecular composition of matter on Earth—from elemental analyses of sediments ^2, 3^ and structural characterization of natural organics ^4^ to 'omics measurements of gene and protein content of natural environmental communities ^5, 6^. Technological advances in computation, data storage, and analytical tools enable this revolution. Alongside these advances is a similar, though often overlooked, revolution in robotics and laboratory automation. High-throughput cultivation in microtiter plates is possible both aerobically and anaerobically, and plate readers can be used to monitor optical density or metabolites using colorimetric assays ^7, 8^. It is also possible to fill microplates with arrays of compounds or serially diluted solutions to simultaneously evaluate the influence of hundreds to tens of thousands of parameters (for example, small-molecule libraries, inorganic ions, carbon sources, or other nutrients) on microbial growth kinetics and metabolism ^7^. Additionally, recent advances in high-throughput genetics can be leveraged (in microbial isolates) to rapidly identify genetic determinants important for fitness in a given growth condition ^9– 11^ ( Figure 2). Importantly, high-throughput assays can be used to quantitatively measure growth and respiratory activity of microbial cultures to define the fitness of a given microbial respiratory metabolism to defined gradients of compounds. Mass spectrometry–based metabolite analysis can give further insights into important metabolic signatures of this activity, and 16S amplicon sequencing can be used to monitor changes in the microbial community in response to these parameters. Subsequent growth-based assays with isolates from a given microbial enrichment culture can be used to measure isolate fitness and isolate gene fitness in response to the same gradients of compounds in which the enrichment was cultivated. Through measuring metabolic activity, microbial community structure, isolate fitness, and gene fitness in the context of gradients of environmentally relevant parameters, we can build models that link gene-, microbe-, and metabolism-specific fitness to environmental context ( Figure 2). Through such workflows, environmental microbiologists now are able to re-array and reconstitute the purified organic and inorganic components of microbial ecosystems at an unprecedented scale and speed.

### Toward multidimensional measurements

Microbes rely on both organic and inorganic cofactors and nutrients and live in complex multidimensional gradients of beneficial, neutral, and toxic compounds ^12, 13^. Microbial niche space is often viewed as an n-dimensional matrix in which antimetabolites, carbon sources, and essential nutrients influence the ability of a microorganism or microbial community to grow and survive ^13, 14^. Thus, only by altering the concentrations of multiple inorganic or organic compounds in a massively parallel, high-throughput cultivation platform can we gain a quantitative bottom-up picture of gene-microbe-metabolism-environment interactions. Several high-throughput approaches developed in the biomedical sciences can be applied to problems in environmental microbiology, including high-throughput screens to identify inhibitory compounds, dose-response microplate assays to quantify the inhibitory potency of compounds, checkerboard synergy assays to evaluate non-linear interactions between compounds, and leave-one-out assays to evaluate formulation potencies ^8, 15^. However, most of the compounds used in the biomedical industry (for example, drugs) are not environmentally relevant. To address this shortfall, we have begun to array metals and other inorganic compounds such as in an “80 metals plate” ( Table 1) to create compound collections that more accurately capture the microbial stressors present in the environment. This arrayed compound collection can be serially diluted and added to microbial cultures to determine inhibitory concentrations such as minimal inhibitory concentration (MIC) or the concentration required to inhibit 50% of control growth (IC _50_). By varying the inoculum, respiratory substrates, and other parameters, experimentalists can gain insights into how other dimensions of the environment influence the inhibitory potency of these inorganic ions on gene-microbe-metabolism fitness.

**Table 1.** 80 metals plate.

Compound name	Stock concentration, mM
Sodium sulfate	1,000
Sodium sulfite	1,000
Sodium selenate	1,000
Sodium selenite	1,000
Sodium perchlorate	1,000
Sodium chlorate	1,000
Sodium silicate	1,000
Sodium nitrate	1,000
Sodium nitrite	100
Sodium phosphate	1,000
Sodium phosphite	1,000
Sodium hypophosphite	1,000
Sodium fluorophosphate	1,000
Sodium arsenate	1,000
Sodium m-arsenite	1,000
Ferric-nitrilotriacetic acid (Ferric-NTA)	10
Zinc-NTA	10
Copper-NTA	10
Potassium chromate	1,000
Sodium molybdate	1,000
Sodium tungstate	1,000
Sodium bromate	1,000
Sodium thiosulfate	1,000
Sodium chloride	2000
Sodium bromide	1,000
Sodium iodide	1,000
Sodium fluoride	1,000
Lithium chloride	1,000
Potassium chloride	1,000
Rubidium chloride	1,000
Cesium chloride	1,000
Magnesium chloride	1,000
Calcium chloride	1,000
Strontium chloride	1,000
Barium chloride dihydrate	10
Chromium(III) chloride	10
Manganese(II) chloride	10
Ferric chloride	100
Cobalt chloride	10
Nickel(II) chloride	10
Copper(II) chloride	10
Zinc chloride	10
Aluminum chloride	10
Cadmium chloride	10
Thallium(I) acetate	10
Cerium(III) chloride	1,000
Europium(III) chloride	100
Ethylenediamine-N,N′- disuccinic acid (EDTA)	500
NTA	500
Chromium-NTA	10
Nickel-NTA	10
Ammonium chloride	1,000
Hydroxylamine hydrochloride	1,000
Vanadium chloride	10
Ferrous ammonium sulfate	10
Beryllium sulfate	1,000
Gallium(III) chloride	100
Lead(II) chloride	10
Sodium cyanide	100
Sodium pyrophosphate	100
Sodium metavanadate	100
Sodium periodate	100
Sodium iodate	100
Sodium thiophosphate	100
Sodium chlorite	100
Sodium hypochlorite	10
Potassium tellurate	1
Silver chloride	1
Potassium hexahydroxoantimonate	10
Gold chloride	1
Mercury chloride	10
Platinum(IV) chloride	10
Palladium(II) chloride	10
Potassium tellurite	10
Boric acid	10
Bismuth chloride	1
Cobalt-NTA	10
Manganese-NTA	10
Cadmium-NTA	10
Aluminum-NTA	10

These compounds are arrayed in a 96-well microplate format that can be serially diluted into other microplate formats for high-throughput cultivation of microbial cultures.

### Microbes know bioinorganic chemistry better than chemists do

**Figure 3.**
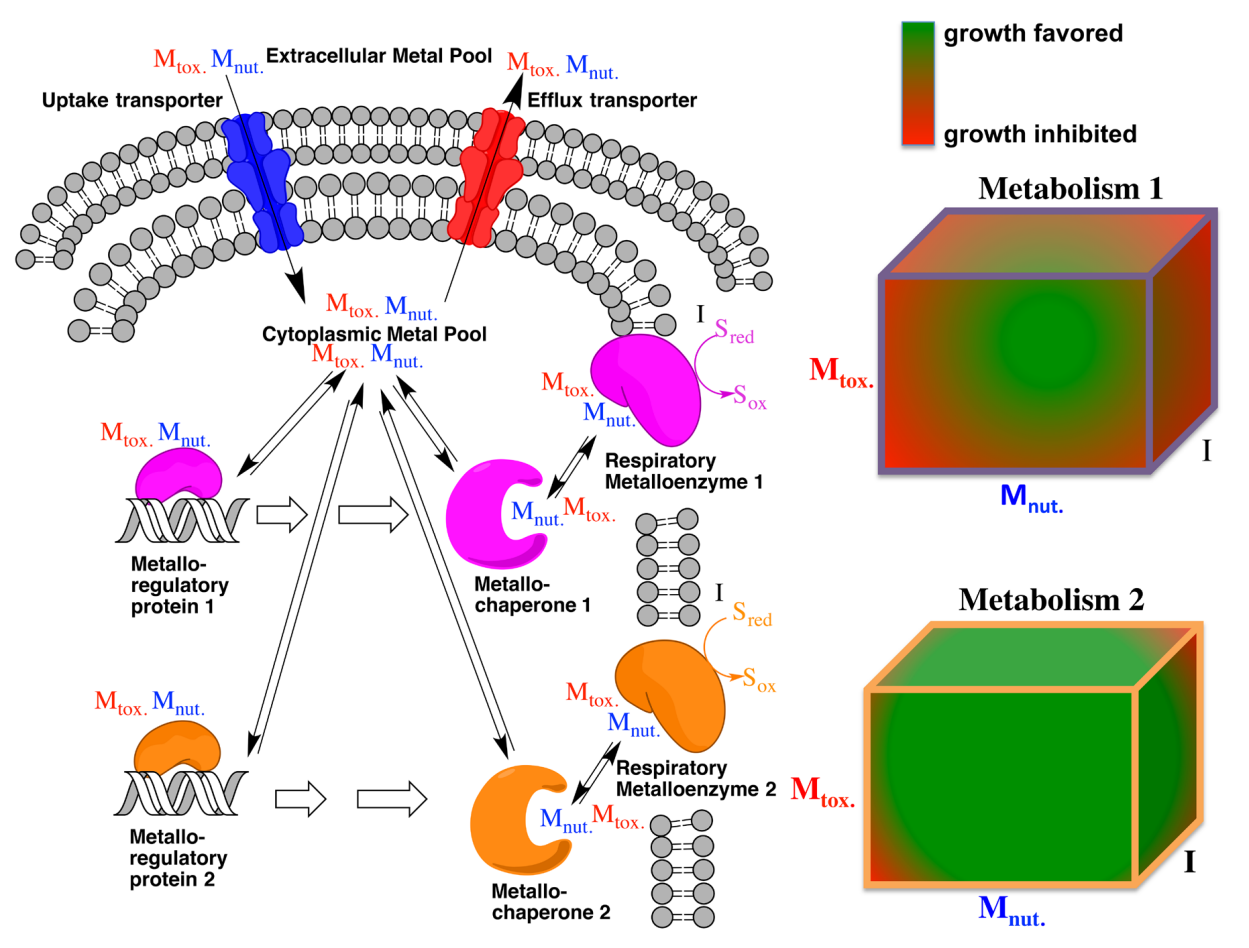
Toxic metals (M _tox._) interfere with the metabolism of essential, nutrient metals (M _nut._). The influence of a toxic metal will vary depending on the metabolism. For example, metabolism 1 and metabolism 2 could be aerobic respiration, nitrate reduction, sulfate reduction, and photosynthesis. Similarly, other metals (I) can serve as antimetabolic inhibitors of respiratory enzymes, competing with substrate (S _red_) for binding and turnover to product (S _ox_). Depending on the inhibitory potency of the toxic metal (M _tox._), the requirements of the essential metal (M _nut._), and the inhibitory potency of a respiratory inhibitor (I), different metabolisms will have different environmental ranges in response to metal gradients.

Organic life exists and evolves in a matrix of both organic and inorganic compounds. One indelible mark of this evolutionary history consists of the diverse metallocofactors incorporated into enzymes that enable chemistry impossible for catalysts composed solely of C, H, N, O, P, and S ^16^. High concentrations of metals are toxic to cells, and many metals also serve no catalytic role. Therefore, resistance mechanisms to metals have evolved. Metals are toxic to microorganisms because of their redox activity and because antimetabolic metals can compete with cofactor metals for binding to biological ligands and proteins ^17^. Not surprisingly, microorganisms have evolved mechanisms for coping with metal stress, and these mechanisms vary by microorganism, metabolic state, or metal and are different depending on the metal concentration ^16, 18^. As an example, iron and its interactions with other transition metals and microbial cells are fairly well studied. Iron is an essential metal for a variety of metalloproteins. Under limiting concentrations of iron, other transition metals can interfere with high-affinity iron uptake systems and metalloregulatory proteins ^19^, but at higher concentrations, some transition metals are toxic because of their ability to catalyze the production of reactive oxygen species ^20^. Thus, the mechanism of toxicity and the mechanisms of resistance will be different depending on the concentrations of the metals. Very few studies systematically evaluate metal toxicity under both excess and limiting concentrations of essential metals, but by quantifying the toxicity of larger panels of metals under these conditions, we can obtain “structure-activity” information for inorganic compounds and their toxicity against, for example, uptake and efflux systems ( Figure 3). A microplate array involving compounds such as the “80 metals plate” described in Table 1 could be serially diluted and used to evaluate the toxicity of many metals simultaneously against microbial isolates, enrichments, and pooled transposon mutants. Metal cations and oxyanions with varying ionic radii, charge, and electron affinity will vary in their interaction with different cellular systems. Only by quantifying the inhibitory potency of these metals under the various conditions under which these different cellular systems are important can we gain insights into how these systems have or have not evolved resistance to various metals. Ultimately, the data obtained through such studies will help geomicrobiologists to infer which metals may have been present in the environment in which a microbe evolved and to quantify the geochemical and genetic parameters that limit the growth of a microbial isolate or community in the environment.

## Metal-metabolism interactions

Metal requirements and toxicity are influenced by the metabolic state of a microorganism. Microorganisms can grow with a range of electron donors, carbon sources, and electron acceptors. All of these metabolisms have unique metal requirements and sensitivities to inorganic antimetabolites and toxins. As such, metals can be selective inhibitors or promoters of different metabolisms. For example, zinc can be more inhibitory of bacteria growing under glucose catabolic conditions versus other carbon sources because zinc inhibits key enzymes in glycolysis ^21^. Against respiratory sulfate reduction, monofluorophosphate, molybdate, and perchlorate are all selective inhibitors with varying selectivities, potencies, and modes of inhibition against the central enzymes in the sulfate reduction pathway ^22^. Some redox-active metals are more inhibitory of aerobically growing cells than anaerobic cells because they can reduce oxygen to superoxide and catalyze Fenton chemistry. By quantifying the inhibitory or stimulatory potencies of large panels of inorganic compounds against microbial isolates and enrichments carrying out various metabolic activities selective compounds can be identified and the degree of their selectivity quantified. Quantification of these tipping points will improve biogeochemical reactive transport models that incorporate predictions of microbial metabolic activities.

### Optimism for the future: identifying novel antimetabolites as predictors of ecosystem function and environmental engineering strategies

Multidimensional microbiology is poised to become the norm in the 21st century. Alongside rapidly improving computational and analytical tools, high-throughput microbial physiology will enable massively parallel measurements of microbial fitness in complex gradients of environmentally relevant conditions. Rarefaction curves from genome sequencing datasets imply that the genetic diversity of life is not infinite ^23^, nor is the elemental composition of biosphere. From this perspective, the “infinite and subtle complexity” ^1^ of the natural world has more to do with the fractal complexity of natural gradients, heterogeneous mixtures in soil, complex water currents, and the corresponding conglomerate of microbial activity in this geochemical milieu. Thus, although we may not reach a comprehensive and flawless model of biogeochemical processes on Earth from bottom-up measurements of microbial fitness and physiology, we are likely to greatly increase the resolution of our models through careful, high-throughput experimentation.

## References

[1] Adams D: The More Than Complete Hitchhiker's Guide: Complete & Unabridged.1989 Reference Source

[2] Smith DB Cannon WF Woodruff LG: Geochemical and Mineralogical Data for Soils of the Conterminous United States.U.S. Geological Survey Data Series 801,2013;19:1–26. Reference Source

[3] Ellefsen KJ Smith DB Horton JD: A modified procedure for mixture-model clustering of regional geochemical data. *Appl Geochem.* 2014;51:315–326. 10.1016/j.apgeochem.2014.10.011

[4] Isaacman G Wilson KR Chan AW: Improved resolution of hydrocarbon structures and constitutional isomers in complex mixtures using gas chromatography-vacuum ultraviolet-mass spectrometry. *Anal Chem.* 2012;84(5):2335–42. 10.1021/ac2030464 22304667

[5] Markowitz VM Chen IA Chu K: Ten years of maintaining and expanding a microbial genome and metagenome analysis system. *Trends Microbiol.* 2015;23(11):730–41. 10.1016/j.tim.2015.07.012 26439299

[6] Wang DZ Kong LF Li YY: Environmental Microbial Community Proteomics: Status, Challenges and Perspectives. *Int J Mol Sci.* 2016;17(8): pii: E1275. 10.3390/ijms17081275 27527164 PMC5000673

[7] Carlson HK Stoeva MK Justice NB: Monofluorophosphate is a selective inhibitor of respiratory sulfate-reducing microorganisms. *Environ Sci Technol.* 2015;49(6):3727–36. 10.1021/es505843z 25698072

[8] Tietjen K Drewes M Stenzel K: High throughput screening in agrochemical research. *Comb Chem High Throughput Screen.* 2005;8(7):589–94. 10.2174/138620705774575300 16305356

[9] Wetmore KM Price MN Waters RJ: Rapid quantification of mutant fitness in diverse bacteria by sequencing randomly bar-coded transposons. *mBio.* 2015;6(3):e00306–15. 10.1128/mBio.00306-15 25968644 PMC4436071

[10] Deutschbauer A Price MN Wetmore KM: Evidence-based annotation of gene function in *Shewanella oneidensis* MR-1 using genome-wide fitness profiling across 121 conditions. *PLoS Genet.* 2011;7(11):e1002385. 10.1371/journal.pgen.1002385 22125499 PMC3219624

[11] Skerker JM Leon D Price MN: Dissecting a complex chemical stress: chemogenomic profiling of plant hydrolysates. *Mol Syst Biol.* 2013;9:674. 10.1038/msb.2013.30 23774757 PMC3964314

[12] Emerson D Breznak JA: The response of microbial populations from oil-brine contaminated soil to gradients of NaCl and sodium *p*-toluate in a diffusion gradient chamber. *FEMS Microbiol Ecol.* 1997;23(4):285–300. 10.1111/j.1574-6941.1997.tb00410.x

[13] Eisenhauer N Schulz W Scheu S: Niche dimensionality links biodiversity and invasibility of microbial communities. *Funct Ecol.* 2013;27(1):282–288. 10.1111/j.1365-2435.2012.02060.x

[14] Wimpenny JW: Spatial order in microbial ecosystems. *Biological Reviews.* 1981;56(3):295–342. 10.1111/j.1469-185X.1981.tb00352.x

[15] Arkin MR Ang KK Chen S: UCSF Small Molecule Discovery Center: innovation, collaboration and chemical biology in the Bay Area. *Comb Chem High Throughput Screen.* 2014;17(4):333–42. 10.2174/1386207317666140323133841 24661212

[16] Crichton R: Biological Inorganic Chemistry.1st ed. Amsterdam: Elsevier;2008;1–383. Reference Source

[17] Outten FW Twining BS: Metal homeostasis: an overview.2007 Reference Source

[18] Gadd GM: Metals, minerals and microbes: geomicrobiology and bioremediation. *Microbiology.* 2010;156(Pt 3):609–43. 10.1099/mic.0.037143-0 20019082

[19] Lee JW Helmann JD: Functional specialization within the Fur family of metalloregulators. *Biometals.* 2007;20(3–4):485–99. 10.1007/s10534-006-9070-7 17216355

[20] Braud A Hoegy F Jezequel K: New insights into the metal specificity of the Pseudomonas aeruginosa pyoverdine-iron uptake pathway. *Environ Microbiol.* 2009;11(5):1079–91. 10.1111/j.1462-2920.2008.01838.x 19207567

[21] Ong CL Walker MJ McEwan AG: Zinc disrupts central carbon metabolism and capsule biosynthesis in *Streptococcus pyogenes*. *Sci Rep.* 2015;5:10799. 10.1038/srep10799 26028191 PMC4450579

[22] Carlson HK Kuehl JV Hazra AB: Mechanisms of direct inhibition of the respiratory sulfate-reduction pathway by (per)chlorate and nitrate. *ISME J.* 2015;9(6):1295–305. 10.1038/ismej.2014.216 25405978 PMC4438318

[23] Land M Hauser L Jun SR: Insights from 20 years of bacterial genome sequencing. *Funct Integr Genomics.* 2015;15(2):141–61. 10.1007/s10142-015-0433-4 25722247 PMC4361730

